# Lnc-DC regulates cellular turnover and the HBV-induced immune response by TLR9/STAT3 signaling in dendritic cells

**DOI:** 10.1186/s11658-018-0108-y

**Published:** 2018-09-03

**Authors:** Lifan Zhuang, Jianhua Tian, Xinzhi Zhang, Hong Wang, Chenghui Huang

**Affiliations:** 1 Department of Infectious Disease, the Affiliated Shenzhen Baoan Hospital of Southern Medical University, Shenzhen, 518101 China; 2 Department of Infectious Disease, Shenzhen Baoan District People’s Hospital, No. 118, Xin’an Street, Long Jing er Raod, Shenzhen, 518101 China

**Keywords:** Dendritic cell, Lnc-DC, TLR9/STAT3, HBV

## Abstract

**Background:**

Lnc-DC is a specific group of long non-coding (Lnc) RNAs in dendritic cells (DCs). Its function has been previously studied, and includes roles in dendritic cell differentiation and the progression of some diseases. In this study, we observed the critical role of Lnc-DC in regulating the differentiation, growth, and apoptosis of dendritic cells.

**Methods:**

We first isolated peripheral blood mononuclear cells to culture and induce into DCs, which were then co-cultured with hepatitis B virus (HBV)-secreting HepG2.2.15 cells for the detection of changes in Lnc-DC. The expression levels of TLR9, p-STAT3, and SOCS3 were tested with qPCR and western blot. MTT assays were used to analyze the cell proliferation, cell cycle, and apoptosis. We used ELISA to test the expression of TNF-α, IL-1β, IL-6, IL-12p40, and IFN-γ.

**Results:**

Co-culture with HBV-secreting HepG2.2.15 cells increased the level of Lnc-DC and activated TLR9/STAT3 signaling. The HBV DNA level (IU/ml) was positively correlated with levels of Lnc-DC and TLR9, further demonstrating that Lnc-DC was associated with the immune response of HBV. Lnc-DC was shown to regulate TLR9/STAT3 signaling in dendritic cells. More interestingly, the regulation of Lnc-DC controlled the immune response by reducing the concentration of secreted TNF-α, IL-6, IL-12, and IFN-γ, as well as increasing the IL-1β concentration in dendritic cells.

**Conclusion:**

Lnc-DC is important in regulating the growth, apoptosis, and immune response of dendritic cells mediated by TLR9/STAT3 signaling, and was also activated by HBV. This study provides a previously unidentified mechanism underlying the immune response in dendritic cells.

## Background

Long non-coding RNAs (Lnc-RNAs) are a type of regulatory RNA less than 200 nt in length with no protein-coding functions. One specific Lnc-RNA in dendritic cell (DC) is Lnc-DC. Knockdown of Lnc-DC has been shown to impair DC differentiation in human monocytes [1]. The role of Lnc-DC in the regulation of STAT3 signaling was recently elucidated in coronary artery disease and type 2 diabetes mellitus [2]. In systemic lupus erythematosus, plasma Lnc-DC was identified as a novel biomarker [3]. Lnc-DC overexpression induced the over-maturation of decidual dendritic cells in preeclampsia patients and led to an increase in Th1 cells [4]. All these findings demonstrate the critical role of Lnc-DC in disease occurrence and progression.

Lnc-DC is located on chromosome 17, near the STAT3 gene. Its regulation on STAT3 signaling has been previously reported. Lnc-DC binds to STAT3, prevents dephosphorylation, and stimulates tyrosine phosphorylation [1]. Phosphorylation of STAT3 is crucial for signaling activation and nuclear translocation [5]. This results in overexpression of target genes and the regulation of cell growth, differentiation, and migration.

Toll-like receptor 9 (TLR9) is well expressed in immune cells. The correlation of TLR9 and STAT3 was elucidated in several cellular types. For example, STAT3 signaling is targeted by TLR9, thereby affecting the immunosuppressive activity of myeloid-derived suppressor cells [6]. TLR9 and STAT3 have a synergic effect on promoting the tumor propagation potential of prostate cancer cells [7]. Meanwhile, TLR9 activation induced an anti-inflammatory response in macrophages through the STAT3-dependent pathway [8].

In these lines, we studied the role of Lnc-DC in the growth, apoptosis and HBV-induced immune response of dendritic cells. Growth was inhibited and apoptosis was promoted in dendritic cells after Lnc-DC knockdown. The immune response was negatively regulated with Lnc-DC knockdown. In addition, we found that Lnc-DC knockdown reduced the expression levels of pSTAT3, TLR9, and SOCS3, demonstrating the involvement of TLR9/STAT3 signaling. The hepatitis B virus (HBV) DNA level was regulated by Lnc-DC and TLR9 signaling in dendritic cells. Therefore, this work elucidated the role of Lnc-DC in dendritic cell growth and the immune response, potentially identifying a new mechanism underlying the correlation between Lnc-DC and the immune response in HBV infection.

## Materials and methods

### Isolation of peripheral blood mononuclear cells

Human peripheral blood mononuclear cells (PBMCs) were prepared as previously described [9]. PBMCs were isolated from 10 mL of venous blood using a Ficoll-Paque PLUS centrifuge as previously described [10]. After centrifugation, cells were collected from the interphase layer and washed four times with RPMI 1640 medium. PBMCs (1 × 10^7^ cells/mL) were suspended in RPMI 1640 supplemented with 10% (*v*/v) and FBS was used to induce the generation of dendritic cells.

### Isolation of primary monocytes from PBMCs

Monocytes from Ficoll-isolated PBMCs were resuspended in PBS and incubated in CD14 microbeads for 15 min at 4 °C. The microbead-labeled cells were then resuspended in PBS after centrifugation and isolated by an MS column. The cells labeled with microbeads were washed from the column with PBS; the resultant cells were CD14+ monocytes.

### Induction of dendritic cells from monocytes

Dendritic cells were generated from monocytes in the presence of GM-CSF (50 ng/ml) and IL-4 (100 ng/ml). The cells were cultured for six days in RPMI1640 growth medium supplemented with 10% FBS. Maturation of dendritic cells was promoted with stimulation by 1 μg/ml LPS for 24 h.

### Flow cytometry

Cell-surface molecule expression of the cultured dendritic cells was evaluated by flow cytometry (FC500, Beckman Coulter), using the following fluorochrome-labeled antibodies: mouse anti-human CD86 FITC (BD Pharmingen) and mouse anti-Human CD83 APC (eBioscience). CXP software from Beckman Coulter was used for the analyses.

### Co-culture of HepG2.2.15 and dendritic cells

The co-culture of HepG2.2.15 and dendritic cells was conducted in a transwell system (Corning). HepG2.2.15 cells were seeded in plate and dendritic cells were grown in inserts. The ratio of HepG2.2.15 to dendritic cells was 2.8:1. Both were maintained in RPMI1640 medium supplemented with 10% FBS.

### Real-time PCR

Total RNA extraction was performed using TRIzol reagent (Life Technologies) according to the manufacturer’s instructions. Two micrograms of total RNA extracted from dendritic cells was subjected to reverse transcription (RT). The cDNA was synthesized using a one-step RT-PCR kit from Takara. SYBR Green (Toyobo) RT-PCR amplification and real-time fluorescence detection were performed using an ABI 7300 real-time PCR thermal cycle instrument (ABI, USA), according to the supplied protocol. The relative gene expression was calculated by the ∆∆Ct method and the relative expression levels were normalized to that of endogenous GAPDH. The primers used were as follows: H-TLR9-F: CGGTTTGATCTGGCTGGACT, H-TLR9-R: AGGCCAGGTAATTGTCACGG; H-SOCS3-F: TGGTCACCCACAGCAAGTTT, H-SOCS3-R CTGTCGCGGATCAGAAAGGT; and Lnc-DC-F: CAGCCTTCCTCCTCCTGTGA, Lnc-DC-R: CAGCCTTCCTCCTCCTGTGA.

### Western blotting

A total of 2 μg of cell lysate was loaded into each lane of a 10% polyacrylamide gel, then blotted onto a polyvinylidene difluoride (PVDF) membrane. After blocking with PBST containing 5% nonfat dry milk, the membrane was incubated with specific primary antibodies against p-Stat3, Stat3, TLR9, and SOCS3. All antibodies were purchased from Cell Signaling Technologies. Peroxidase-linked IgG (Life Technologies) was used as the secondary antibody. These proteins were visualized with an ECL western blotting detection kit (Amersham Biosciences).

### Viral production and infection

A lentivirus expressing Lnc-DC-shRNA (shLnc-DC) was produced and purified using the BLOCK-iT Inducible H1 Lentiviral RNAi System (Life Technologies). An shRNA with a scramble sequence was used to generate the control virus. All the manufacturer’s procedures were strictly followed. Dendritic cells in each well were infected with 2 × 10^6^ pfu of virus. The analysis was conducted three days after infection.

### MTT assay

MTT was used to evaluate the proliferation of dendritic cells after the downregulation of Lnc-DC. Briefly, cells were incubated with MTT for at least 4 h to produce formazan. When the formazan was completely dissolved by SDS-HCl, the absorbance was measured at 570 nm with a Universal Microplate Reader (Bio-Tek instruments), and OD (MLB-treated group)/OD (blank control group) was calculated.

### Analysis of cell cycle phase by flow cytometry

Forty-eight hours following infection with lenti-shLnc-DC, dendritic cells were resuspended in PBS twice before fixation by dropwise addition to 95% precooled ethanol. Prior to analysis, the cells were warmed, centrifuged at 450 g for 5 min, resuspended twice in PBS, then stained with propidium iodide PI (containing RNase A at 50 μg/ml) at room temperature in the dark for 30 min. The DNA content was analyzed by flow cytometry using the CellQuest program (Becton-Dickinson).

### Annexin V/7-AAD staining

Cells were washed twice with staining buffer, and then resuspended in Annexin V binding buffer. FITC-Annexin V and the 7-AAD staining solution were added and incubated with cells for 15 min at room temperature in the dark. We used 488 nm excitation and measured the fluorescence emission near 530 nm (FITC channel) for Annexin V and > 670 nm (PE channel) for 7-AAD by flow cytometry.

#### Enzyme-linked immunosorbent assay

The inflammatory factors in the cell culture supernatant after Lnc-DC silencing were detected by an ELISA kit with quantitative measurements, according to the manufacturer’s recommendations. The inflammatory factors included TNF-α, IL-1β, IL-6, IL-12, and IFN-γ (CUSABIO).

### Statistical analysis

All data were presented as mean ± SD. The band density in the western blot analysis was measured with Image J software (NIH). Statistical significance was determined by unpaired Student’s t-test using SigmaPlot Software (Systat Software, San Jose, CA, USA). A value of *p* < 0.05 was considered to be significant.

## Results

### Coculture of dendritic and HepG2.2.15 cells activated TLR/STAT3 signaling

**Fig. 1:**
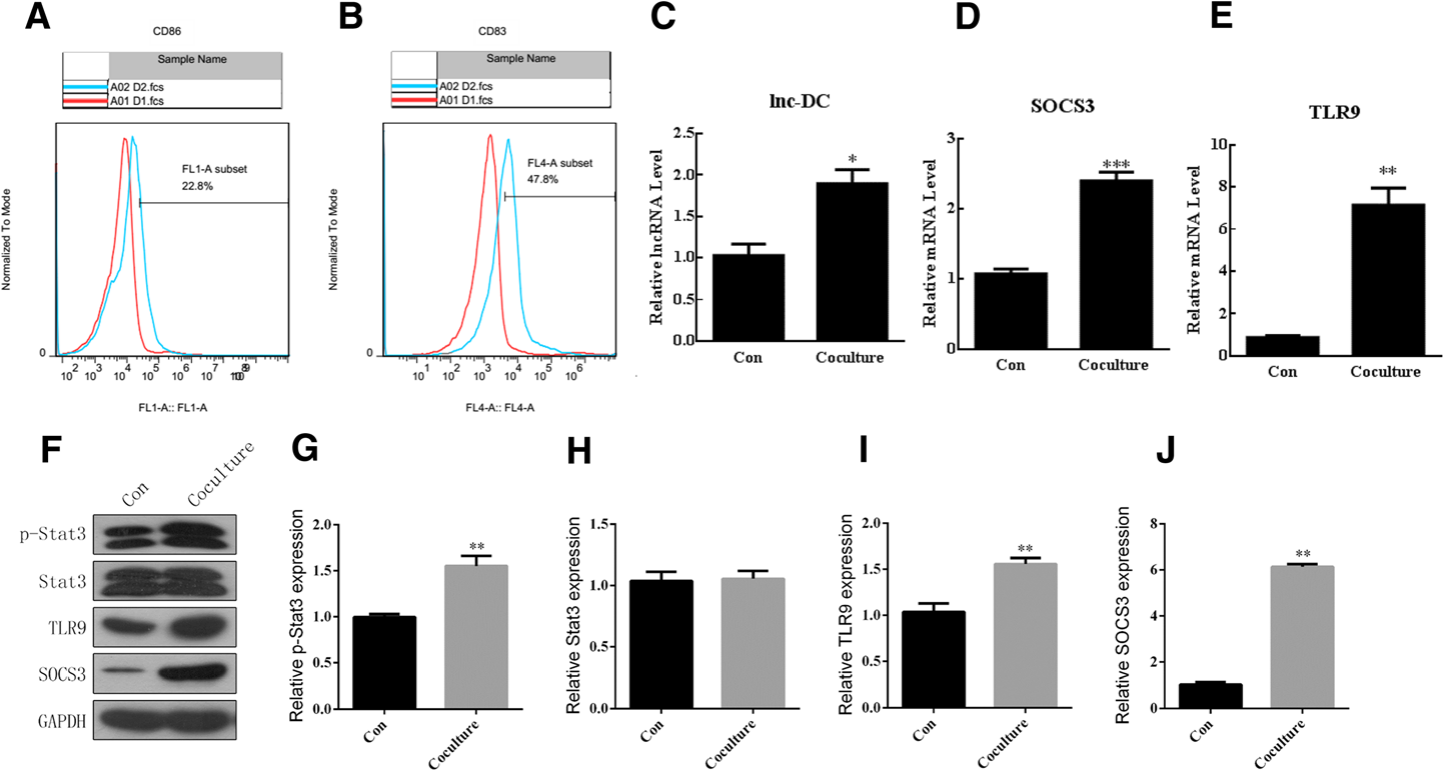
Coculture of dendritic and HepG2.2.15 cells **a**, **b** Characterization of dendritic cells by flow cytometry against CD83 and CD86. **c**-**e** Co-culture with HepG2.215 cells increased mRNA levels of Lnc-DC, SOCS3, and TLR9. **f**-**j** Co-culture with HepG2.215 cells increased the protein levels of p-Stat3, TLR9, and SOCS3. The statistical analysis was determined by unpaired Student’s t test. *p < 0.05, **p < 0.01, ***p < 0.001

Dendritic cells were induced from PBMCs and characterized by flow cytometry against CD83 and CD86 (Fig. 1a, b). After coculture with HepG2.2.15 cells, TLR/STAT3 signaling was activated, as evidenced by quantitative real-time PCR demonstrating that the Lnc-DC, SOCS3, and TLR9 mRNA levels increased (Fig. 1c, d and e). The activation of TLR/STAT3 signaling was confirmed by western blotting, in that the levels of p-STAT3, TLR9, and SOCS3 increased in dendritic cells cocultured with HepG2.2.15 cells (Fig. 1f, g, h, i and j). Thus, TLR/STAT3 signaling was activated in dendritic cells when cocultured with cells secreting HBV.

### Silencing Lnc-DC regulated TLR/STAT3 signaling in dendritic cells

**Fig. 2:**
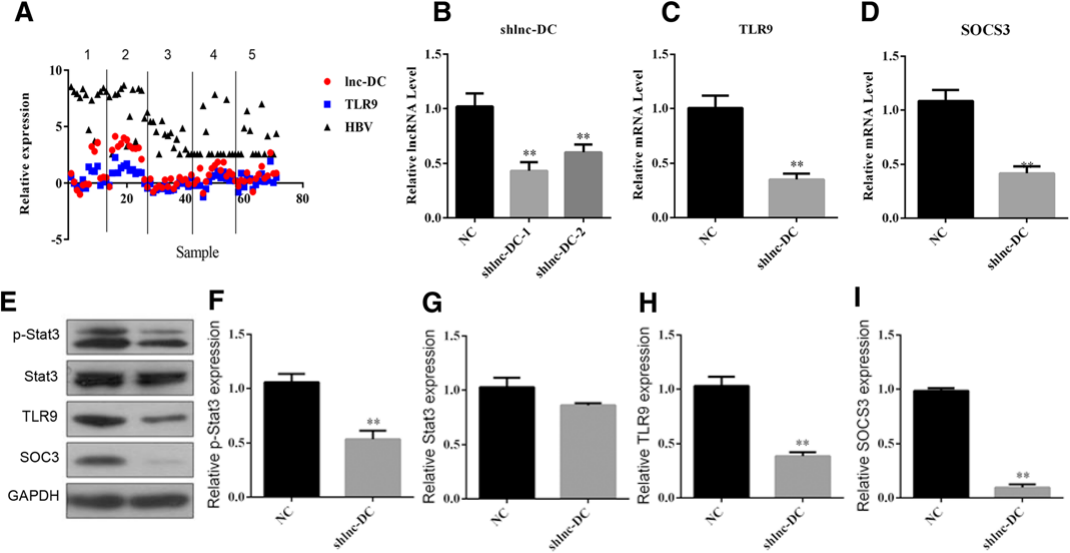
Expression of Lnc-DC downregulation in dendritic cells **a** The mRNA levels of Lnc-DC and TLR9 were positively correlated with the level of HBV DNA. Lnc-DC downregulation in dendritic cells decreased the level of Lnc-DC **b** TLR9 **c** and SOCS3 **d** **e**-**i** Lnc-DC downregulation decreased the protein expression of p-Stat3, TLR9, and SOCS3. The statistical analysis was determined by unpaired Student’s t test. **p < 0.01

Next, we measured the correlations among Lnc-DC, HBV, and TLR9 in the PBMCs of six different groups: non-infected people, infected people in the immunotolerant phase, infected people in the immune clearance phase, infected people in the recovery phase, infected people in the relapse phase, and infected people with hepatitis B cirrhosis. The mRNA levels of Lnc-DC and TLR9 were positively correlated with the level of HBV DNA (Fig. 2a). In order to elucidate the role of Lnc-DC in dendritic cells, cells were infected with lenti-shRNA-Lnc-DC. The Lnc-DC mRNA level was reduced to ~ 50% of that of the control group, showing the efficiency of lenti-shRNA-Lnc-DC (Fig. 2b). With the downregulation of Lnc-DC, the mRNA level of TLR9 and SOCS3 significantly decreased (Fig. 2c, and d, *p* < 0.01). Similarly, western blotting indicated that the levels of p-STAT3, TLR9, and SOCS3 decreased in shLnc-DC infected dendritic cells (Fig. 2e, f, g, h, and i, *p* < 0.01). This indicates that Lnc-DC regulated TLR/STAT3 signaling in dendritic cells.

### Silencing of Lnc-DC affected dendritic cell proliferation

**Fig. 3:**
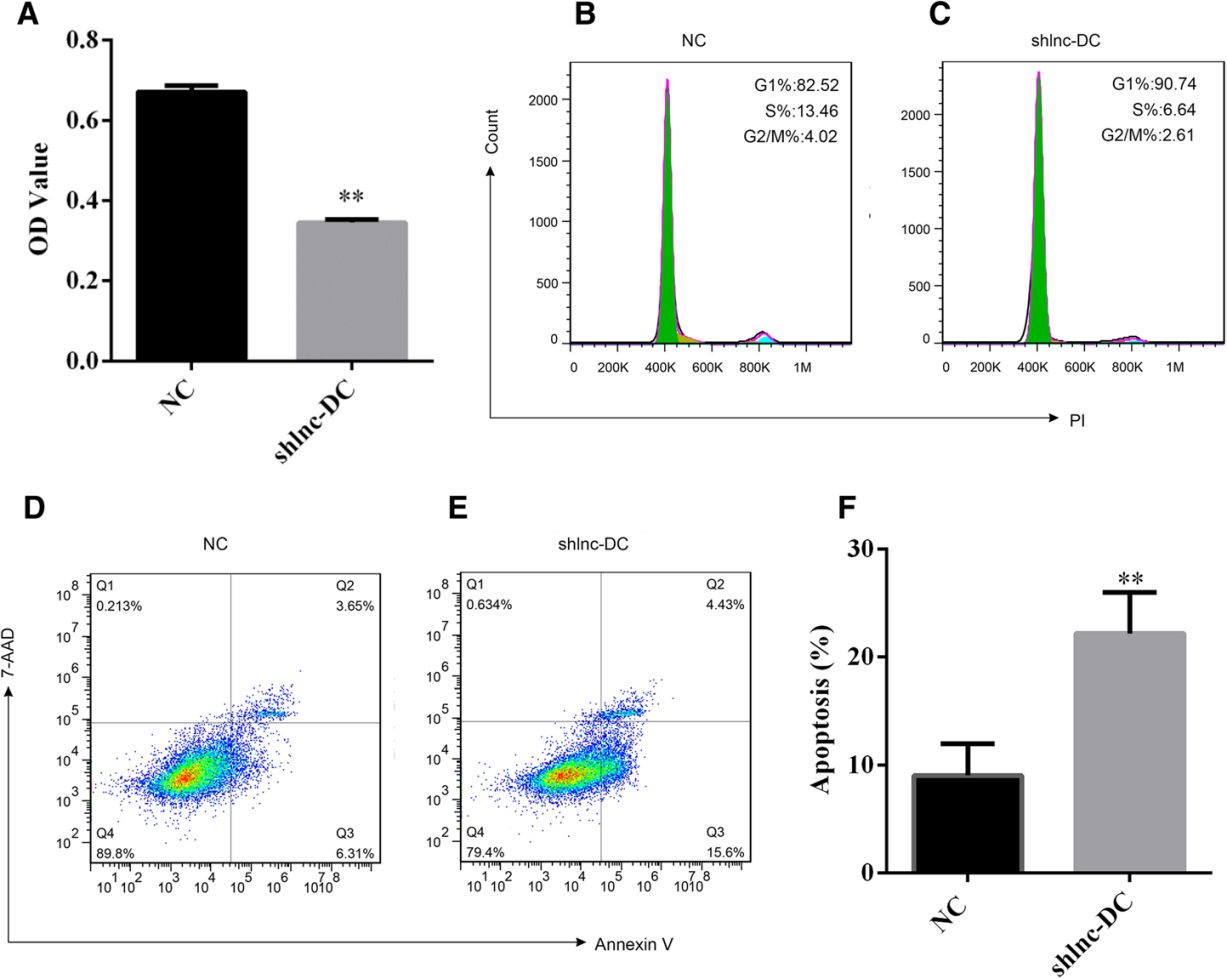
Lnc-DC downregulation affects dendritic cells’ function **a** Lnc-DC silencing inhibited proliferation in dendritic cells. **b**, **c** Lnc-DC silencing affected the cell cycle in dendritic cells. **d**-**f** The ratio of apoptotic cells was drastically increased by 7-AAD/Annexin V staining. The statistical analysis was determined by unpaired Student’s t test. **p < 0.01

Next, we determined whether Lnc-DC downregulation affected the proliferation of dendritic cells. An MTT assay indicated that Lnc-DC downregulation could impair the proliferation of dendritic cells (Fig. 3a). To further demonstrate whether or not the expression of Lnc-DC regulated dendritic cell death, we analyzed the alteration to the cell cycle in correlation with the downregulation of Lnc-DC. The cell cytometry analysis showed that the ratio of cells at S or G2/M phase decreased while the ratio of cells at G1 phase increased (Fig. 3b, and c). This indicated that Lnc-DC downregulation arrested fewer dendritic cells at G1 phase. 7-AAD/Annexin V staining showed that the ratio of apoptotic cells was significantly increased in the shLnc-DC group (*p* < 0.01, Fig. 3d, e and f). These data demonstrate that Lnc-DC regulated the proliferation and apoptosis of dendritic cells.

### Lnc-DC silencing regulated the immune response in dendritic cells

**Fig. 4:**
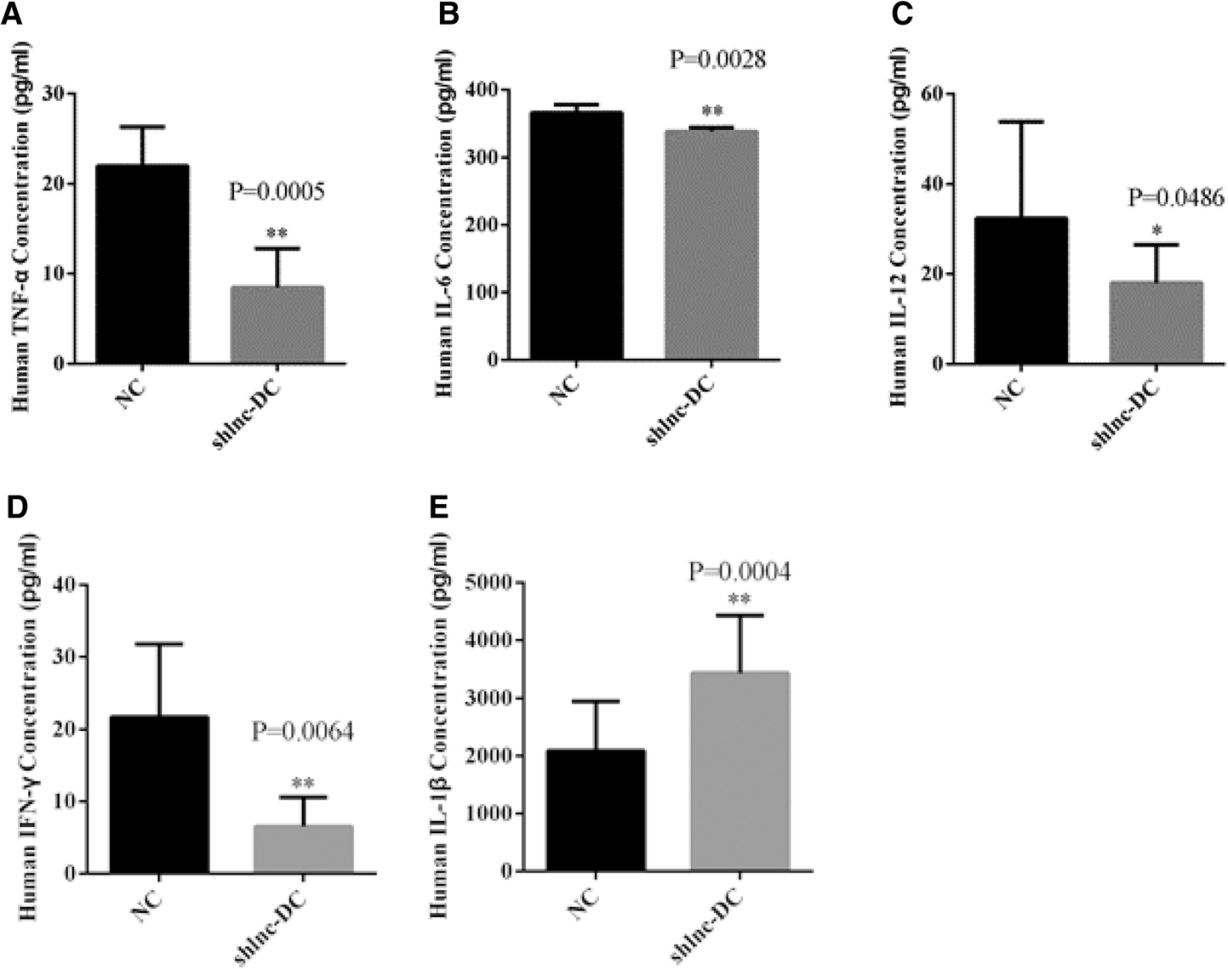
Lnc-DC downregulation affects immune response Lnc-DC silencing regulated the immune response through decreasing the concentration of TNF-α **a** IL-6 **b** IL-12 **c** and IFN-γ **d** as well as increasing the IL-1β concentration. The statistical analysis was determined by unpaired Student’s t test. *p < 0.05, **p < 0.01

Since inflammation is an important feature of dendritic cells, we asked whether Lnc-DC silencing could affect the immune response. The ELISA data indicate that Lnc-DC silencing significantly decreased the concentration of TNF-α (*p* < 0.001, Fig. 4a). Similarly, we also observed a reduction in the concentration of IL-6 (*p* < 0.01, Fig. 4b), IL-12 (*p* < 0.05, Fig. 4c), and IFN-γ (*p* < 0.01, Fig. 4d). The decrease of IL-6 in the shLnc-dc group was minimal. By contrast, we observed a drastic increase in the concentration of IL-1β with the downregulation of Lnc-DC (Fig. 4e). In summary, these data show that Lnc-DC can regulate inflammation in dendritic cells.

## Discussion

In this study, we demonstrated the role of Lnc-DC in regulating the growth, apoptosis, and immune response in dendritic cells through TLR9/STAT3 signaling. HBV DNA was also associated with TLR9/STAT3 signaling. Therefore, we proposed a new mechanism underlying the cellular turnover in dendritic cells and the immune response by HBV.

One interesting finding of our study was that the co-culture of HepG2.215 and dendritic cells activated TLR9/STAT3 signaling and the expression level of TLR9 was positively correlated with HBV DNA in PBMCs from hepatitis B patients. HepG2.215 is a human hepatic cell line that has been widely used in HBV production for antiviral research [11]. Thus, the activation of TLR9/STAT3 signaling in the co-culture system indicated that HBV induced TLR9/STAT3 signaling. The stimulation of dendritic cells leads to a boost in endogenous T-cell immunity. Nevertheless, the effect of HBV on dendritic cells is still not clear [12]. The exhaustion of chronic HBV-specific T cells makes it difficult to boost HBV-specific T-cell immunity and the action of HBV on dendritic cells is difficult to understand. Many studies have shown that inflammatory cytokines are involved in the immune response. For example, IL-1 was proven to support the activation of immune response induction [13]. IL-6 and TGF-β participate in immune regulation of dendritic cells [14]. Thus, we detected the concentrations of IL-1β, TNF-α, IL-6, IL-12, and IFN-γ. The decreased concentrations of these inflammatory cytokines showed that Lnc-DC may play a significant role in the regulation of the immune response. More interestingly, the concentration of IL-1β increased when Lnc-DC was inhibited, the reasons for which can be explored in the future. Through this study, we linked HBV-infected dendritic cells via Lnc-DC regulation with TLR9/STAT3 signaling. This is the first piece of evidence to show the role of Lnc-RNAin the HBV-induced immune response of dendritic cells, as well as its underlying mechanism. However, we did not identify the subtypes of dendritic cells in which HBV led to the activation of TLR9/STAT3 signaling [15–17]. In order to more accurately describe the correlation between dendritic cells, Lnc-DC, and HBV, characterization of dendritic cells will be a future research direction. In addition, the mechanisms by which HBV increases the expression of Lnc-DC should also be further studied.

We found that Lnc-DC regulated TLR9/STAT3 signaling in dendritic cells. The correlation between Lnc-RNAs and STAT3 signaling has been described in multiple tumor cells [18–20]. In this study, we elucidated the role of Lnc-DC in dendritic cells mediated by TLR9/STAT3 signaling. Lnc-DC knockdown induced inactivation of TLR9/STAT3, which led to decreased proliferation and increased apoptosis. This potentially indicates that an alteration of cellular turnover in dendritic cells is due to TLR9/STAT3 inactivation. Thus, the downstream effects of TLR9/STAT3 need to be investigated to reveal the underlying mechanism. ERK signaling is a potential downstream effector in controlling the immune response mediated by TLR9/STAT3 [21]. This may also be associated with inactivation of NF-κB signaling and activation of apoptotic pathways. All of these need to be further investigated.

## Conclusion

This study investigated the immune response mechanism of Lnc-DC, and the findings suggest that TLR9/STAT3 signaling participates in the immune response, which was activated by HBV. This study identifies a previously unidentified mechanism underlying the immune response in dendritic cells.

## Abbreviations

DC: Dendritic cells
HBV: Hepatitis B virus
Lnc: Long non-coding
Lnc-RNAs: Long non-coding RNAs
PBMCs: Peripheral blood mononuclear cells
PVDF: Polyvinylidene difluoride
shLnc-DC: Lnc-DC-shRNA
